# Tourniquet Use and Anesthesiology Considerations in Patients With Hypermobile Ehlers-Danlos Syndrome: Balancing Orthopedic Needs With Anesthetic Safety

**DOI:** 10.7759/cureus.105190

**Published:** 2026-03-13

**Authors:** Kaitlyn Unterman, Laith Fada, Kathleen A Clark, Julie Cherolis

**Affiliations:** 1 Medicine, Alabama College of Osteopathic Medicine, Dothan, USA; 2 Anesthesiology, North Baldwin Infirmary, Bay Minette, USA

**Keywords:** compression neuropathy, hypermobile ehlers-danlos syndrome (heds), ischemia-reperfusion injury, orthopedic anesthesia, pneumatic tourniquet

## Abstract

Hypermobile Ehlers-Danlos syndrome (hEDS) is a hereditary connective tissue disorder characterized by generalized joint hypermobility, soft tissue fragility, chronic pain, and autonomic dysfunction. Although major vascular complications are uncommon in hEDS, affected individuals frequently undergo orthopedic procedures, including ligament reconstructions, tendon repair, and joint stabilization, due to recurrent dislocations and musculoskeletal injury. Consequently, careful evaluation of pneumatic tourniquet use in this patient population is warranted.

Pneumatic tourniquets are widely used in extremity orthopedic surgery to reduce blood loss and improve visualization. However, their effects on peripheral nerve integrity, pain outcomes, and soft tissue injury in patients with hEDS remain poorly understood. These concerns are particularly relevant in the context of regional anesthesia, where chronic pain syndromes and autonomic dysfunction may complicate perioperative analgesia and postoperative neurologic assessments.

This narrative review examines the pathophysiology of hEDS and explores anesthetic and orthopedic considerations related to tourniquet use in this patient population. While tourniquets offer clear intraoperative advantages, patients with hEDS may be at increased risk for skin injury, compression neuropraxia, exaggerated ischemia-reperfusion pain, and delayed postoperative recovery. In the absence of evidence-based guidelines, tourniquet use in patients with hEDS should involve shared decision-making among anesthesiologists, surgeons, and patients. Individualized discussions addressing potential risks, alternative techniques, and modifiable tourniquet parameters, such as pressure levels, duration, and padding placement, may help mitigate perioperative complications.

## Introduction and background

Hypermobile Ehlers-Danlos syndrome (hEDS) is a hereditary connective tissue disorder characterized by generalized joint hypermobility, musculoskeletal instability, chronic pain, and autonomic dysfunction, including postural orthostatic tachycardia syndrome (POTS) [1,2]. Unlike classical and vascular Ehlers-Danlos syndrome (EDS) subtypes, hEDS lacks an identified monogenic etiology and is diagnosed clinically based on standardized criteria incorporating joint hypermobility, characteristic musculoskeletal manifestations, and supportive family history or exclusion of alternative diagnoses [1,3].

Although hEDS is not typically associated with major vascular rupture, affected individuals experience substantial musculoskeletal morbidity. Chronic joint pain, recurrent subluxations, dislocations, and soft-tissue injuries are common and frequently result in orthopedic consultation and surgical intervention [3,4]. Castori et al. described a characteristic disease trajectory consisting of an early hypermobility phase, followed by a prolonged pain-predominant phase, during which many patients pursue operative management due to functional impairment and joint instability [3].

Pneumatic tourniquets are routinely employed in extremity orthopedic surgery to improve visualization, reduce intraoperative blood loss, and enhance procedural efficiency [5]. However, tourniquet application is associated with well-described complications in the general population, including nerve compression injury, ischemia-reperfusion pain, muscle injury, and skin or soft-tissue damage [6,7]. In patients with hEDS, underlying connective tissue fragility, baseline neuropathic symptoms, and autonomic dysfunction may amplify susceptibility to these adverse effects [8-10].

Despite increasing recognition of hEDS within both anesthetic and orthopedic practices, formal guidance addressing tourniquet use in this population remains absent. This narrative review integrates anesthetic and orthopedic perspectives to examine the pathophysiologic considerations, potential risks, and perioperative management strategies relevant to pneumatic tourniquet use in patients with hEDS, with emphasis on individualized decision-making and risk mitigation.

Methods

This is a narrative review examining anesthetic and orthopedic considerations related to pneumatic tourniquet use in patients with hEDS. A narrative methodology was selected due to the limited availability of hEDS-specific data and the heterogeneous nature of the existing literature, which precludes systematic comparison or statistical pooling.

A literature search was performed using PubMed/MEDLINE, Google Scholar, and the Cochrane Library. English-language articles were included without strict date limitations. Search terms included combinations of “hypermobile Ehlers-Danlos syndrome,” “Ehlers-Danlos syndrome,” “joint hypermobility syndrome,” “pneumatic tourniquet,” “surgical tourniquet,” “limb occlusion pressure,” “regional anesthesia,” “perioperative management,” “neuropathic pain,” and “ischemia-reperfusion injury.” Reference lists of relevant articles were manually reviewed to identify additional studies. Articles were selected based on relevance to hEDS pathophysiology, orthopedic or anesthetic use of pneumatic tourniquets, tourniquet-related complications, and perioperative management strategies. Given the scarcity of literature specifically addressing tourniquet use in hEDS, studies from the general orthopedic and anesthetic populations and other Ehlers-Danlos syndrome subtypes were included when physiologically relevant and identified as extrapolated evidence.

No quantitative or statistical synthesis was performed. A meta-analysis was not feasible due to the limited number of hEDS-specific studies, heterogeneity in study designs, and variability in reported outcomes. The available evidence consists primarily of physiologic studies, retrospective analyses, case series, and expert consensus statements, which are not amenable to formal statistical pooling. Findings were therefore qualitatively synthesized and organized thematically, with emphasis on practical, modifiable perioperative considerations to support individualized clinical decision-making.

## Review

Pathophysiology relevant to tourniquet use

Hypermobile Ehlers-Danlos syndrome is characterized by disordered connective tissue architecture affecting skin, fascia, vasculature, peripheral nerves, and musculoskeletal structures. Although hEDS lacks a single identifiable genetic mutation, abnormalities in collagen organization and extracellular matrix integrity result in impaired force distribution and reduced tissue resilience [11,12]. These features have direct implications for pneumatic tourniquet application. Effective arterial occlusion requires circumferential compression across multiple tissue layers. In patients with hEDS, uneven force transmission beneath the tourniquet cuff may lead to focal pressure gradients, increasing the risk of skin injury, subcutaneous hemorrhage, and delayed tissue recovery, even at pressures considered safe in the general population [13,14].

Neurovasculature vulnerability further compounds risk. Capillary fragility predisposes patients to hematoma formation, while baseline neuropathic symptoms and proprioceptive deficits suggest underlying peripheral nerve susceptibility [15,16]. Tourniquet-induced ischemia followed by reperfusion may exacerbate nerve injury through oxidative stress, inflammatory activation, and microvascular dysfunction [17]. Central sensitization, a well-recognized feature of hEDS, may amplify pain responses and prolong postoperative recovery following relatively minor mechanical or ischemic insults [18]. 

Neurologic risks associated with tourniquet use in hEDS

Tourniquet-related nerve injury represents the most frequently reported serious complication of pneumatic tourniquet use. In the general surgical population, nerve compression and ischemia may result in transient sensory deficits, neuropraxia, or, less commonly, prolonged motor weakness [19,14].

In patients with hEDS, these risks may be magnified due to baseline peripheral nerve vulnerability and chronic neuropathic pain syndromes [16,18]. Compression-related neurologic symptoms may persist longer than expected, complicating postoperative neurologic assessment and rehabilitation. Tourniquet deflation may also provoke exaggerated pain responses and autonomic instability, particularly in patients with dysautonomia or central sensitization, further challenging postoperative pain control [18,20]. Although severe neurologic complications remain uncommon, the combination of connective tissue fragility, altered pain perception, and autonomic dysfunction supports a cautious and individualized approach to tourniquet application in this population.

Skin and soft tissue vulnerability

Skin and soft tissue complications associated with tourniquet use include bruising, subcutaneous hematoma formation, pressure-related skin injury, and delayed wound healing. Patients with hEDS frequently exhibit capillary fragility and easy bruising despite normal coagulation profiles, increasing susceptibility to compression-related injury beneath tourniquet cuffs [6,15]. Even in the absence of overt skin breakdown or necrosis, subtle soft tissue injury may contribute to prolonged postoperative pain and delayed functional recovery. These effects may not be adequately captured by standard outcome measures used in the general population and may be amplified in patients with connective tissue disorders due to impaired tissue resilience and heightened pain sensitivity [13].

Orthopaedic rationale for tourniquet use

Patients with hEDS frequently require orthopedic intervention due to recurrent joint instability, soft-tissue failure, and high rates of failure following primary surgical repair. Ligamentous laxity and altered joint biomechanics complicate both initial surgical management and revision procedures, which are common in this population [4,21].

Pneumatic tourniquets are commonly employed to improve visualization and reduce intraoperative blood loss, particularly in procedures involving compromised soft tissue planes. However, in patients with hEDS, the same factors that necessitate surgical intervention, such as connective tissue fragility and altered biomechanics, may also increase susceptibility to pressure-related injury beneath the tourniquet cuff [13]. Consequently, the routine use of tourniquets in this population warrants careful scrutiny.

Procedure location plays a critical role in risk stratification. Upper-extremity surgeries involving the elbow, forearm, or hand carry an increased risk of nerve compression due to the superficial course of major neurovascular structures and limited soft-tissue padding [22]. In contrast, lower-extremity procedures often involve longer operative times, thereby increasing cumulative ischemic exposure, muscle injury, and postoperative pain [17]. These risks may be further amplified during revision surgeries, where scar tissue, distorted anatomy, and prolonged operative duration necessitate extended tourniquet inflation times.

Joint hypermobility and ligamentous laxity also increase the risk of intraoperative subluxation or dislocation, particularly when a tourniquet restricts repositioning or requires sustained limb elevation. Meticulous attention to padding, limb alignment, and frequent reassessment of limb position is therefore essential to minimize iatrogenic injury [23].

From an orthopedic perspective, tourniquet use in patients with hEDS should not be considered routine or mandatory. Alternative strategies, including tourniquet-free techniques, meticulous hemostasis, or intermittent tourniquet deflation, may be appropriate in selected cases. When tourniquets are deemed necessary, minimizing inflation pressure, limiting duration, and avoiding unnecessary proximal placement are prudent strategies supported by general tourniquet safety principles.

Ultimately, the orthopaedic rationale for tourniquet use in hEDS must be individualized, balancing procedural complexity and operative efficiency against patient-specific risk factors. Close collaboration with anesthesia is essential to optimize intraoperative conditions while minimizing the potential for tourniquet-related complications.

Anesthetic considerations in hEDS

Anesthetic management plays a central role in mitigating perioperative and tourniquet-related risk in patients with hEDS. A thorough preoperative evaluation should include assessment of joint stability, identification of autonomic dysfunction, and review of chronic pain history and current medications. Baseline hemodynamic measurements are particularly valuable in patients with POTS, as they may help predict intraoperative blood pressure lability and guide anesthetic management [1,2]. Preoperative optimization may include fluid loading, vasoactive support planning, and consultation with cardiology for severe autonomic dysfunction.

Vascular access can be challenging in this population due to venous fragility and a propensity for infiltration or hematoma formation, mirroring risks associated with circumferential compression devices. Ultrasound-guided peripheral or central venous access can improve success and minimize trauma. Although standard coagulation profiles are typically normal in hEDS, easy bruising and bleeding tendencies are common across EDS subtypes and should be considered during perioperative planning [6,16]. Minimizing repeated needle attempts and using smaller gauge catheters when possible can reduce soft tissue injury.

Intraoperatively, tourniquet pressures should be minimized to the lowest effective level, and extra padding should be applied to prevent skin and soft tissue injury. Positioning should avoid hyperextension, valgus stress, or excessive traction on vulnerable joints. Using gel pads, foam supports, and adjustable positioning devices can protect skin, ligaments, and joint capsules. Frequent checks for pressure points or joint malalignment are recommended, especially for prolonged procedures [5].

Hemodynamic monitoring should be tailored to the patient’s autonomic profile. Continuous noninvasive blood pressure monitoring may be sufficient for minor procedures, but invasive arterial monitoring should be considered for prolonged or high-risk surgeries, particularly in patients with labile blood pressure or a history of syncope. Electrocardiography and pulse oximetry remain standard, and capnography is mandatory during general anesthesia and conscious sedation [24]. Awareness of exaggerated responses to tourniquet inflation and deflation, including sudden hypertension or tachycardia, is critical, and readiness with short-acting vasoactive agents is advised.

Both general and regional anesthesia are feasible but require careful consideration of joint stability and hemodynamic goals. For general anesthesia, short-acting induction agents and titratable maintenance anesthetics help manage labile blood pressure. Opioids should be used cautiously, balancing analgesia with the risk of hypotension or delayed recovery. Regional anesthesia can be beneficial for pain control, but meticulous technique is required to avoid nerve injury due to joint hypermobility or fragile connective tissue. Local anesthetic dosing may need adjustment in areas of reduced tissue resistance [5].

Postoperatively, multimodal analgesia including acetaminophen, nonsteroidal anti-inflammatory drugs (NSAIDs) if not contraindicated, gabapentinoids, and local infiltration techniques are recommended to manage chronic pain and minimize opioid-related complications. Wound and soft tissue monitoring is essential to detect hematoma, bruising, or delayed healing. Early, gentle, supervised mobilization helps prevent joint injury while optimizing functional recovery. Patients with hEDS often experience chronic pain due to nociceptive and neuropathic mechanisms arising from central sensitization [12,17]. Opioid management in this population requires particular caution because opioids themselves can paradoxically worsen pain through opioid-induced hyperalgesia (OIH), a phenomenon in which repeated opioid exposure triggers neuroplastic changes that increase pain sensitivity rather than reduce it [18].

Tourniquet-specific considerations in hEDS

Tourniquet-related anesthetic complications include heightened ischemia-reperfusion pain, delayed resolution of compression-related nerve symptoms, and prolonged postoperative neuropathic pain [18,22]. Tourniquet deflation may provoke significant discomfort and autonomic instability, particularly in patients with baseline dysautonomia or pain sensitization.

In hEDS, pneumatic tourniquet use may increase the risk of nerve injury, prolonged neuropraxia, skin breakdown, and subcutaneous hematoma formation [14,24]. Although compartment syndrome and rhabdomyolysis are rare, their risk may be heightened in fragile tissues with prolonged ischemia, especially at high cuff pressures. Guidelines for other EDS subtypes recommend avoiding tight tourniquets during blood collection in patients with pressure-induced hyperalgesia [25].

Individualized tourniquet pressure based on limb occlusion pressure (LOP) is a promising strategy. Tuncali et al. demonstrated effective upper-extremity hemostasis using LOP with a 20 mmHg safety margin, achieving mean pressures of 171.5 ± 23.7 mmHg, substantially lower than the traditional 250 mmHg standard [20]. Lower extremity pressures of SBP + 75-120 mmHg provided adequate fields with fewer complications than SBP + 150 mmHg [26].

Perioperative hemostatic optimization remains important. Strategies include antifibrinolytics (tranexamic or mefenamic acid) or desmopressin to reduce bleeding and hematoma formation [16,27]. Additional monitoring may include near-infrared spectroscopy to detect early neural ischemia and vigilance for tourniquet-induced hypertension [21,22].

Risk mitigation strategies

When tourniquet use is unavoidable in patients with hEDS, perioperative risk mitigation should be approached in a structured, phase-specific manner, focusing on modifiable factors and informed by both available evidence and expert consensus (Table 1). Preoperative planning is critical, as patients with EDS experience significantly higher surgical complication rates compared with the general population. Multidisciplinary coordination among anesthesia, surgery, pain management, and, when appropriate, genetics teams allows for individualized risk stratification and anticipatory planning. Baseline assessments of neurologic function, skin integrity, and joint stability should be documented preoperatively to facilitate postoperative assessment and distinguish preexisting conditions from new complications. Informed consent represents a key mitigation strategy, with explicit discussion of the increased risks of hematoma formation, impaired wound healing, chronic postoperative pain, and potential neurovascular injury, thereby supporting shared decision-making and appropriate expectation setting. 

**Table 1:** Risk mitigation strategies for tourniquet use in patients with hEDS hEDS: Hypermobile Ehlers-Danlos syndrome, EDS: Ehlers-Danlos syndrome, LOP: Limb occlusion pressure

Perioperative phase	Risk factor	Mitigation strategy	Rationale	Strength of evidence	Key references
Preoperative	Elevated surgical complication rates in EDS	Multidisciplinary preoperative planning when tourniquet use is anticipated	EDS patients demonstrate markedly higher complication rates compared with the general population	Observational clinical data	Yonko et al. [5]
Preoperative	Unrecognized baseline neurologic or skin abnormalities	Document baseline neurologic exam, skin integrity, and joint stability	Facilitates differentiation between baseline symptoms and postoperative injury	Expert consensus	Shirley et al. [26]
Preoperative	Inadequate patient expectations	Explicit informed consent addressing the elevated risk of hematoma, poor wound healing, chronic pain, and neurovascular injury	Improves shared decision-making and postoperative satisfaction	Expert consensus	Yonko et al. [5]
Intraoperative	Excessive tourniquet pressure	Individualize pressure using LOP rather than fixed thresholds	Lower pressures reduce nerve and soft tissue injury without compromising hemostasis	High-quality physiologic and clinical studies	Tuncali et al. [20]
Intraoperative	Increased tissue compression and pain	Use wide tourniquet cuffs when feasible	Wider cuffs achieve arterial occlusion at lower pressures and reduce pain	Biomechanical and clinical evidence	Masri et al. [28]
Intraoperative	Prolonged ischemia time	Minimize tourniquet inflation duration and avoid unnecessary reinflation	Reduces ischemia-reperfusion injury and neuropraxia	Expert opinion; extrapolated evidence	Estebe et al. [22]
Intraoperative	Autonomic instability (dysautonomia)	Close hemodynamic monitoring; anticipate tourniquet-induced hypertension	Tourniquet inflation may increase BP by ≥30% above baseline	Physiologic studies; hEDS extrapolation	Pyati et al. [21]
Intraoperative	Undetected neural ischemia	Consider near-infrared spectroscopy adjacent to at-risk nerves	Allows early detection of ischemia prior to clinical deficits	Emerging clinical evidence	Pyati et al. [21]
Postoperative	Delayed recognition of nerve injury or compartment syndrome	Perform early and serial neurologic examinations	Enables timely diagnosis of rare but limb-threatening complications	Expert consensus	Estebe et al. [22]
Postoperative	Central sensitization and chronic pain	Implement opioid-sparing multimodal analgesia (lidocaine, ketamine, dexmedetomidine)	Targets neuroinflammation and pain sensitization common in hEDS	Emerging evidence	Ramírez-Paesano. et al. [27]
Postoperative/regional anesthesia	Concern for nerve injury with blocks	Use ultrasound-guided peripheral nerve blocks when indicated	Case series (21 blocks in 16 patients) and individual case reports show no block-related complications, supporting safety	Low-level evidence (case series and case reports)	Neice et al. [23], Patzkowski et al. [25]
Perioperative	Hemostatic abnormalities	Consider antifibrinolytics or desmopressin in selected patients	May mitigate bleeding tendency and bruising in hEDS	Limited guideline-based evidence	Lindsay et al. [16], Kosho et al. [19]
Perioperative	Undiagnosed high-risk EDS subtype	Genetics consultation for atypical features or high-risk history	Identifies patients with vascular or visceral rupture risk	Guideline-based	Shirley et al. [26]
Extrapolated evidence	Pressure-induced hyperalgesia (non-hEDS EDS subtypes)	Avoid tight tourniquets; favor cautious pressure selection	Supports broader vulnerability of connective tissue disorders	Guideline-based extrapolation	Kosho et al. [19]

Intraoperatively, tourniquet-related variables represent the most modifiable contributors to morbidity in hEDS. Individualization of tourniquet pressure using limb occlusion pressure rather than fixed pressure thresholds is supported by physiologic and clinical data demonstrating effective hemostasis at substantially lower pressures and reduced nerve and soft-tissue injury. Wider tourniquet cuffs are preferred as they achieve arterial occlusion at lower pressures and are associated with less pain and tissue damage. Limiting tourniquet inflation duration and avoiding unnecessary reinflation may further reduce ischemia-reperfusion injury and prolonged neuropraxia. Tourniquet inflation is associated with a progressive rise in blood pressure (tourniquet-induced hypertension), while deflation can cause sudden hypotension due to reactive hyperemia and washout of ischemic metabolites. Given these hemodynamic fluctuations, close cardiovascular monitoring is recommended during both inflation and deflation. Adjunctive monitoring modalities, such as near-infrared spectroscopy positioned adjacent to vulnerable nerve tissue, may offer early detection of neural ischemia, although current evidence remains limited.

Postoperative mitigation strategies emphasize early recognition and proactive management of neurologic and pain-related complications. Serial neurologic examinations are recommended to facilitate prompt identification of nerve injury or compartment syndrome, particularly in the setting of connective tissue fragility and altered pain perception. Multimodal, opioid-sparing analgesic regimens, including agents such as lidocaine, ketamine, and dexmedetomidine, may be particularly beneficial in hEDS by targeting central sensitization and chronic neuroinflammation. Ultrasound-guided peripheral nerve blocks have been reported as a safe regional anesthesia option in hEDS. A retrospective case series of 21 regional anesthetics in 16 patients with predominantly hypermobility-type EDS reported no block-related complications, and individual case reports describe successful ultrasound-guided lower-extremity nerve blocks without incident, supporting their use when clinically indicated [23,25]. Collectively, these perioperative strategies underscore the importance of individualized, evidence-informed care pathways to mitigate tourniquet-related risk in this vulnerable patient population. 

Limitations and directions for future research

The evidence addressing tourniquet use in patients with hEDS is limited and extrapolated from studies of tourniquet-related complications in the general population. Few investigations explicitly differentiate hEDS from vascular hEDS, despite their markedly distinct risk profiles. Additionally, most available data are derived from retrospective series or case reports with small sample sizes and inconsistent outcome reporting. Neurologic and pain-related complications are particularly difficult to interpret in this population due to the different baseline neuropathic symptoms. Future prospective studies should prioritize standardized reporting, incorporation of patient-reported outcomes, and development of evidence-based consensus recommendations. Controlled trials comparing modified tourniquet strategies with tourniquet-free techniques can help establish optimal practice.

Discussion 

Pneumatic tourniquets remain integral to extremity orthopedic surgery; however, their use is inherently associated with both mechanical and ischemic stress on the tissues distal to the cuff. In general surgical populations, longer inflation times and higher pressures correlate with increased nerve compression and ischemia-reperfusion injury, mechanical deformation of muscles, and nerve dysfunction secondary to high-pressure gradients at cuff edges [23,14]. In hEDS patients whose connective tissues and extracellular matrix are inherently fragile, these basic tourniquet-related pathophysiologic mechanisms likely amplify cumulative stress on muscle, nerve, and skin, warranting cautious and individualized application. 

Tourniquet-induced nerve injury is the most common serious local complication, ranging from mild transient sensory changes to persistent neuropraxia or motor weakness; although complete recovery tends to be the end result, timelines can be unpredictable and prolonged [29, 30]. In non-EDS cohorts, nerve palsy remains the most frequently reported tourniquet complication. Despite the physiologic rationale supporting increased risk in hEDS, direct evidence specific to this population remains sparse, but the basic principles derived from general tourniquet science suggest that lowering pressure, shortening inflation times, optimizing cuff width, and individualizing application based on LOP are strategies to reduce ischemic and mechanical stress. Prospective clinical studies evaluating specific tourniquet protocols in hEDS are needed to establish evidence-based practice. Until such data is available, balancing operative benefit with mitigation of perioperative risk is crucial.

Beyond neurologic manifestations, tourniquet use also affected soft-tissue healing. Delayed wound healing, increased bruising, and exaggerated postoperative pain responses are commonly reported, and these might not be adequately captured by standard metrics used for the general population [3,15]. The absence of obvious nerve damage or skin lesions and necrosis should not be interpreted as the absence of tourniquet-induced harm, as subtle tissue injury contributes to longer recovery times, increased pain management requirements, and overall dissatisfaction with surgical outcomes [13].

Despite the elevated risk supported by strong physiologic rationale, direct evidence guiding tourniquet use specifically in hEDS remains limited. Current practice extrapolates from general tourniquet science combined with an appreciation of the unique vulnerabilities inherent to connective tissue disorders [31]. Strategies such as tailoring cuff pressure, minimizing inflation, and critically evaluating whether a tourniquet meaningfully improves operative efficiency in each case represent risk-mitigation approaches. Prospective studies examining patient-centered outcomes and complications in hEDS are needed to define guidelines. Until that is available, balancing surgical benefit against cumulative perioperative stress remains essential in optimizing care for this challenging patient population [28].

## Conclusions

Pneumatic tourniquet use in patients with hEDS requires heightened perioperative awareness due to underlying connective tissue fragility, baseline neuropathic symptoms, and autonomic dysfunction. While tourniquets offer clear intraoperative advantages, their mechanical and ischemic effects may disproportionately impact this population, increasing the risk of pain amplification, soft-tissue injury, and prolonged neurologic symptoms. In the absence of evidence-based guidelines, tourniquet application in hEDS should be individualized, emphasizing minimized pressure and duration, meticulous padding and positioning, and multidisciplinary decision-making. Future prospective studies are needed to establish optimized tourniquet strategies that balance surgical efficacy with patient safety in this vulnerable group.
